# AIDS-related opportunistic illnesses and early initiation of HIV care remain critical in the contemporary HAART era: a retrospective cohort study in Taiwan

**DOI:** 10.1186/s12879-018-3251-1

**Published:** 2018-07-28

**Authors:** Chun-Yuan Lee, Yu-Ting Tseng, Wei-Ru Lin, Yen-Hsu Chen, Jih-Jin Tsai, Wen-Hung Wang, Po-Liang Lu, Hung-Chin Tsai

**Affiliations:** 1 Division of Infectious Diseases, Department of Internal Medicine, Kaohsiung Medical University Hospital, Kaohsiung Medical University, No.100, Tzyou 1st Road, Kaohsiung, 807 Taiwan; 2 Graduate Institute of Medicine, Kaohsiung Medical University, Kaohsiung, Taiwan; 3 Center for Infectious Disease and Cancer Research (CICAR), Kaohsiung Medical University, Kaohsiung, Taiwan; 4 Division of Infectious Diseases, Department of Medicine, Kaohsiung Veterans General Hospital, 386 Ta-Chung 1st Rd., Kaohsiung, 813 Taiwan; 5 Sepsis Research Center, Graduate Institute of Medicine, College of Medicine, Graduate Institute of Medicine, Kaohsiung Medical University, Kaohsiung, Taiwan; 6 School of Medicine, College of Medicine, Kaohsiung Medical University, Kaohsiung, Taiwan; 7 Department of Biological Science and Technology, College of Biological Science and Technology, National Chiao Tung University, Hsin Chu, Taiwan; 8 Tropical Medicine Center, Kaohsiung Medical University Hospital, Kaohsiung, Taiwan; 9 Tropical Medicine Research Center, College of Medicine, Kaohsiung Medical University, Kaohsiung, Taiwan; 10 Department of Laboratory Medicine, Kaohsiung Medical University Hospital, Kaohsiung, Taiwan; 11 Faculty of Medicine, School of Medicine, National Yang-Ming University, Taipei, Taiwan

**Keywords:** AIDS, HIV, Late presentation, Opportunistic illness

## Abstract

**Background:**

No study has reported the epidemiology of AIDS-related opportunistic illnesses (AOIs) in patients with newly diagnosed HIV infection in Taiwan in the past decade. Understanding the current trends in AOI-related morbidity/mortality is essential in improving patient care and optimizing current public health strategies to further reduce AOIs in Taiwan in the era of contemporary highly active antiretroviral therapy (HAART).

**Methods:**

Eligible patients were evaluated at two referral centers between 2010 and 2015. The patients were stratified by date of diagnosis into three periods: 2010–2011, 2012–2013, and 2014–2015. The demographics, HIV stage at presentation according to the United States CDC 2014 case definition, laboratory variables, and the occurrence of AOIs and associated outcomes were compared among the patients. Logistic regression and Cox regression were respectively used to identify variables associated with the occurrence of AOIs within 90 days of HIV enrollment and all-cause mortality.

**Results:**

Over a mean observation period of 469 days, 1264 patients with newly diagnosed HIV with a mean age of 29 years and mean CD4 count of 275 cells/μL experienced 394 AOI episodes in 290 events. At presentation, 37.7% of the patients had AIDS; the frequency did not significantly differ across groups. The overall proportion of AOIs within the study period was 21.0%, and no decline across groups was observed. The majority of AOIs (91.7%) developed within 90 days of enrollment. All-cause and AOI-related mortality did not significantly differ across groups. Throughout the three study periods, AOIs remained the main cause of death (47/56, 83.9%), especially within 180 days of enrollment (40/42, 95.2%). A CD4 cell count of < 200 cells/μL at presentation was associated with increased adjusted odds of an AOI within 90 days [adjusted odds ratio, 40.84; 95% confidence intervals (CI), 12.59–132.49] and an elevated adjusted hazard of all-cause mortality (adjusted hazard ratio, 11.03; 95% CI, 1.51–80.64).

**Conclusions:**

Despite efforts toward HIV prevention and management, early HIV care in Taiwan continues to be critically affected by AOI-related morbidity and mortality in the era of contemporary HAART. Additional targeted interventions are required for the earlier diagnosis of patients with HIV.

**Electronic supplementary material:**

The online version of this article (10.1186/s12879-018-3251-1) contains supplementary material, which is available to authorized users.

## Background

Prior to the era of highly active antiretroviral therapy (HAART) and effective antimicrobial therapy, AIDS-related opportunistic illnesses (AOIs) caused high morbidity and mortality rates in patients with HIV [1]. In the late 1990s, AOI-related morbidity and mortality began to decline substantially in both resource-limited [2–4] and resource-rich regions [5–7] with the routine use of antimicrobial prophylaxis against *Pneumocystis jiroveci* pneumonia and *Mycobacterium avium* complex, widespread use of potent HAART, development of critical care [8], and enhanced retention across the HIV treatment cascade.

Although chronic non-AIDS-related diseases are emerging as a major cause of morbidity and mortality [9–11], AOIs remain common at presentation for HIV screening or at the first HIV-related medical visit in both developing (33.6–48.0%) [12, 13] and developed countries (15.3–53.8%) [14–18]. AOIs are also the leading cause of hospitalization [19, 20] and death [10, 21] in patients with HIV, and still present a considerable challenge for health care systems attempting to effectively manage HIV.

HIV infection has been a reportable disease in Taiwan since 1984, and was diagnosed in 33,423 patients by the end of 2016. Of these patients, 15,418 developed AIDS and 5569 died [22]. Several strategies have been adopted to improve the HIV care continuum in Taiwan, including free access to HAART and management of AOIs since 1997; initiation of HAART at higher CD4 count thresholds (CD4 < 200 cells/μL in 2006, < 350 cells/μL in 2010, < 500 cells/μL in 2013, and in all patients, regardless of CD4 count in 2016); implementation of the HIV case management program since 2007; and development of LGBT community health centers since 2010. Although interventions have been implemented to combat the HIV epidemic in Taiwan [23], the number of HIV infections reported annually continue to increase [22], and only a few studies have investigated the epidemiology of AOI-related morbidity and mortality in patients with HIV about 10 years ago [24–26]. The epidemiology of AOI-related morbidity and mortality in patients with HIV has not been reported in Taiwan in the past decade. Moreover, both newly diagnosed patients and those with existing HIV diagnoses have been included in most reports [24, 25]. The combination of these two populations in a study design does not reflect the epidemiology of AOIs for newly diagnosed patients after seeking medical care for HIV. Understanding the current trends in AOI-related morbidity and mortality in patients newly diagnosed HIV is essential in improving patient care and optimizing current public health strategies.

This study was conducted at the two largest HIV-designated centers in southern Taiwan, and the current trends in AOI-related morbidity and mortality were analyzed in patients that were newly diagnosed as having HIV between January 1, 2010 and December 31, 2015.

## Method

### Study design, setting, and data source

This study had a retrospective cohort design; it was conducted at Kaohsiung Medical University Chung-Ho Memorial Hospital (KMUH) and Kaohsiung Veterans General Hospital (VGHKS), the two largest referral centers for the treatment of patients with HIV in southern Taiwan. The health care staff at both KMUH and VGHKS have extensive experience in the treatment of patients with HIV and have been actively involved in voluntary counseling and testing (VCT) for HIV since 1997. The study was approved by the institutional review boards of both participating hospitals. The medical records of all eligible patients were reviewed by trained staff, who retrieved the patients’ age, sex, date of the first HIV-related medical visit, HIV diagnosis through active surveillance, presence of risk factors for HIV-1 acquisition, serum CD4+ T-cell counts, HIV viral load at presentation, HIV stage at presentation, and AOIs with associated outcomes.

### Participants

We screened patients who presented at both hospitals for HIV-related care for the first time between January 1, 2010 and December 31, 2015. Those who had received follow-up care at both hospitals were counted once. Because both hospitals were designated to provide care for patients with HIV who were incarcerated, we included only patients who visited each hospital’s outpatient or inpatient department in person. We excluded patients without a new diagnosis, those younger than 15 years, and those seen only once as outpatients and were thus lost to follow-up during the observation period. We stratified the enrolled patients by the date of their initial HIV-related visit into three periods: period 1, from January 1, 2010 to December 31, 2011; period 2, from January 1, 2012 to December 31, 2013; and period 3, from January 1, 2014 to December 31, 2015. We compared demographic and clinical variables, comorbidities, baseline laboratory results, AOI events, and associated outcomes of the patients among the groups.

### Definitions

No consensus exists regarding the definition of a new diagnosis of HIV [27]; however, eligible patients received their initial HIV diagnosis at one of the study hospitals or had been diagnosed elsewhere but referred to one of the aforementioned hospitals within 1 month and had available medical records [28]. The date of enrollment was defined as the date of the first visit at which the patient sought HIV care at either hospital. The date of an AOI event was defined as the date of the first visit at which the patient sought care for an AOI. The final patient observation was at 6 months after each study period, death, or the final outpatient visit or hospitalization for patients who were lost to follow-up, whichever occurred first. HIV diagnoses through active surveillance by VCT, military screening, screening at blood donation, prenatal screening, and screening at methadone clinics were included as initial diagnoses. Baseline CD4 cell count, HIV viral load, and laboratory test results were measured within 6 months of enrollment [14] or as close as possible to the date of HIV diagnosis. The stage of HIV infection at presentation was scaled in the range of 0–3 following the United States Centers for Disease Control and Prevention (CDC) 2014 case definition for HIV infection [29]. Acute HIV infection was defined as acute retroviral syndrome with a negative or indeterminate Western blot in the presence of a positive p24-antigen or HIV-1 RNA test, either detectable or as a documented seroconversion, and with or without symptoms during the preceding 90 days [30]. AOI-related mortality was defined as any death attributable to an AOI.

AOIs were defined according to the United States CDC 1993 AIDS case definition [31] with the addition of *Penicillium marneffei* infection, which is a common AOI in Southeast Asia, southern China, and Taiwan [19, 32]. If patients experienced more than one AOI episode during the same outpatient visit or hospitalization, the episodes were recorded as a single event. AOI episodes occurring during different outpatient visits or hospitalizations were recorded as separate events if no symptoms had been observed at previous outpatient visits or hospitalizations, and the associated laboratory results and outcomes were recorded sequentially.

### Outcomes

Trends in AOI-related morbidity and mortality during the three study periods were the primary outcomes of interest. The AOI-related morbidity and mortality observed in each study period were then stratified by the interval from enrollment in HIV care. The identification of predictors of AOIs occurring within 90 days of enrollment for HIV care and of all-cause mortality was the secondary objective.

### Statistical analysis

Categorical variables among the three subgroups were compared using the χ^2^ or Fisher’s exact test, and continuous variables were compared using analysis of variance. The probabilities of AOIs stratified by study period and all-cause mortality stratified by CD4 cell count at presentation were estimated using Kaplan–Meier survival curves and log-rank testing. In the univariable analysis, CD4 cell count at presentation was used instead of HIV stage at presentation due to the marked collinearity between these two variables. Variables associated with the occurrence of any AOIs within 90 days of enrollment for HIV care were identified using logistic regression analysis, and the factors associated with all-cause mortality were identified using Cox regression analysis. All variables in the univariable analysis were selected for subsequent multivariable analysis. The effects of each variable were estimated using odds ratios (ORs) or hazard ratios (HRs) with 95% confidence intervals (CIs). All tests were two-tailed, and *p* < 0.05 was considered significant. Statistical analyses were performed using SPSS version 22.0 (IBM Corp., Armonk, NY, USA).

## Results

### Characteristics of study participants

Of the 2124 patients screened, 1950 met the inclusion criteria in terms of outpatient or inpatient department visits. A total of 686 patients were excluded because they were not newly diagnosed (*n* = 591), were not followed up for the second time (*n* = 94), or were younger than 15 years (*n* = 1). Of the remaining 1264 enrolled patients, 395 (31.2%) were included in period 1, 471 (37.3%) in period 2, and 398 (31.5%) in period 3.

The baseline demographic characteristics and comorbidities of the three groups are summarized in Table 1. The mean (±standard deviation, SD) duration of observation was 469 (±255) days, 98.3% of the patients were men, and the mean (±SD) age at presentation was 29 (±9) years. The mean (±SD) CD4 cell count at presentation was 275 (±205) cells/μL, and 38.8% of participants had a CD4 cell count of < 200 cells/μL. The frequency of diagnoses through active HIV surveillance was not significantly different among the three enrollment periods (*P* = 0.12), but those made during VCT significantly increased from 33.4% in period 1 to 43.0% in period 3 (*P* = 0.01). The three main routes of HIV transmission were homosexual or bisexual contact (87.2%), heterosexual contact (11.2%), and drug injection (1.1%). The distribution of HIV stages at presentation was not different among the three periods. The overall prevalence of AIDS at presentation according to the United States CDC 2014 case definition for HIV infection [29] (AOIs or CD4 cell count < 200 cells/μL) was 37.7% [38.5% in period 1, 38.4% in period 2, and 36.2% in period 3 (*P* = 0.74)]. The duration of observation, sex, mean age at presentation, comorbidities, mean CD4 cell count at presentation, and mean viral load at presentation did not differ significantly among the patients included in the three study periods.

**Table 1:** Demographics, HIV-related variables, and prevalence of AOIs of newly diagnosed HIV patients enrolled at two medical centers between January 1, 2010 and December 31, 2015

	Total (2010–2015) *N* = 1264	Period 1 (2010–2011) *N* = 395	Period 2 (2012–2013) *N* = 471	Period 3 (2014–2015) *N* = 398	*P*-value
Mean observation period, days (SD)	469 (255)	464 (247)	476 (256)	469 (261)	0.76
Male, n (%)	1242 (98.3)	388 (98.2)	460 (97.7)	394 (99.0)	0.33
Mean age at presentation, years (SD)	29.43 (9.50)	29.87 (10.18)	29.61 (9.81)	28.79 (8.33)	0.25
Diagnoses of HIV due to active HIV surveillance, n (%)	653 (49.7)	188 (45.6)	257 (52.7)	208 (50.3)	0.12
VCT	500 (39.6)	132 (33.4)	197 (41.8)	171 (43.0)	0.01
Military screening	124 (9.8)	47 (11.9)	50 (10.6)	27 (6.8)	0.04
Screening at blood donation	28 (2.3)	9(2.1)	10 (2.3)	9 (2.2)	0.99
Prenatal screening	1 (0.1)	0 (0.0)	0 (0.0)	1 (0.3)	0.34
HIV transmission route, n (%)					
Homosexual	1031 (81.6)	317 (80.3)	376 (79.8)	338 (84.9)	0.11
Heterosexual	141 (11.2)	56 (14.2)	51 (10.8)	34 (8.5)	0.04
Bisexual	71 (5.6)	17 (4.3)	39 (8.3)	15 (3.8)	0.006
IDU	14 (1.1)	3 (0.8)	4 (0.8)	7 (1.8)	0.32
Unknown	7 (0.6)	2 (0.5)	1 (0.2)	4 (1.0)	0.29
Comorbidities, n (%)					
Chronic kidney disease	6 (0.5)	1 (0.3)	2 (0.4)	3 (0.8)	0.58
DM	23 (1.8)	10 (2.5)	6 (1.3)	7 (1.8)	0.38
Solid tumor	11 (0.9)	5 (1.3)	5 (1.1)	1 (0.3)	0.26
Autoimmune disease	7 (0.6)	3 (0.8)	3 (0.6)	1 (0.3)	0.60
Mean CD4 count at presentation, cells/L (SD)^a^	275 (205)	271 (198)	264 (208)	293 (207)	0.09
Subgroup of CD4 cell count at presentation, n (%)^a^					
CD4 cell count < 200 cells/μL	491 (38.8)	154 (39.0)	189 (40.1)	148 (37.2)	0.67
CD4 cell count 200–499 cells/μL	612 (48.4)	199 (50.4)	226 (48.0)	187 (47.0)	0.62
CD4 cell count ≥500 cells/μL	161 (12.7)	42 (10.6)	56 (11.9)	63 (15.8)	0.07
Mean VL (log) (SD)^a^	4.79 (0.83)	4.78 (0.90)	4.80 (0.85)	4.78 (0.73)	0.96
HIV VL > 100,000 copies/mL, n (%)^a^	482 (38.1)	160 (40.5)	179 (38.0)	143 (35.9)	0.50
HBsAg seropositivity, n (%)	117 (9.3)	44 (11.1)	41 (8.7)	32 (8.0)	0.28
HCV seropositivity, n (%)	19 (4.8)	8 (1.7)	15 (3.8)	42 (3.3)	0.033
HIV stage at presentation by 2014 CDC definition [29], n (%)					
Stage 0 (Acute HIV)	100 (7.9)	38 (9.6)	34 (7.2)	28 (7.0)	0.32
Stage 1 (CD4 count ≥500 cells/μL)	141 (11.2)	37 (9.4)	51 (10.8)	53 (13.3)	0.20
Stage 2 (CD4 count 200–499 cells/μL)	546 (43.2)	168 (42.5)	205 (43.5)	173 (43.5)	0.95
Stage 3 (AOIs or CD4 cell count < 200 cells/μL)	477 (37.7)	152 (38.5)	181 (38.4)	144 (36.2)	0.74
AOIs during observation period, n (%)					
Patients with AOI(s) at HIV presentation	233 (18.4)	77 (19.5)	87 (18.5)	69 (17.3)	0.74
Patients who developed AOI(s) within study period	266 (21.0)	89 (22.5)	99 (21.0)	78 (19.6)	0.60
Mortality during observation period, n (%)					
AOI-related mortality	47 (3.7)	13 (3.3)	22 (4.7)	12 (3.0)	0.38
All-cause mortality	56 (4.4)	17 (4.3)	27 (5.7)	12 (3.0)	0.15

*Abbreviations*: *AIDS* acquired immune deficiency syndrome, *AOI* AIDS-defining opportunistic illness, *DM* diabetes mellitus, *HBsAg* hepatitis B surface antigen, *HCV* hepatitis C virus, *HIV* human immunodeficiency virus, *IDU* intravenous drug users, *PY* person-year, *SD* standard deviation, *VCT* voluntary counseling and testing, *VL* viral load

^a^Assayed within 6 months of enrollment

### Trends in AOI-related morbidity and risk of AOIs within 90 days of enrollment for HIV care

**Fig. 1:**
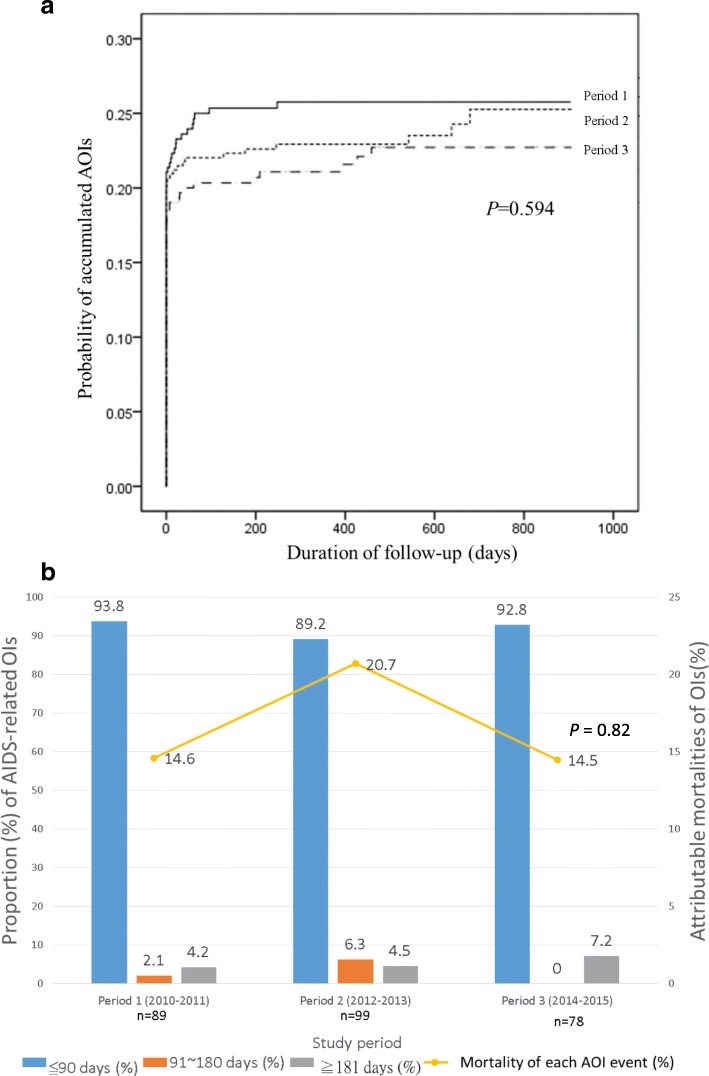
Analysis of AOI-related morbidity. **a** Kaplan–Meier curves of accumulated AOIs in patients with newly diagnosed HIV infection in the three study periods. The probabilities of accumulated AOI events did not significantly differ among the three study periods (log-rank test, *P* = 0.594). In all three periods, the probability of an AOI event increased sharply during the first 3 months following initial HIV care. **b** Stratification of AOIs by interval since enrollment and mortality in each enrollment period. Of the 290 AOI events in the observation period, 96 (33.1%) occurred in period 1, 111 (38.3%) in period 2, and 83 (28.6%) in period 3; 266 of the 290 events developed within 90 days of HIV enrollment (91.7%). The proportion of events that occurred within 90 days did not differ across groups after stratification by interval after the initiation of HIV care (*P* = 0.105). The overall mortality of each AOI event was 16.9% [14.6% in period 1, 20.7% in period 2, and 14.5% in period 3 (*P* = 0.817)]

During 1622 person-years of observation, 394 AOI episodes occurred in 290 events, with 1.36 episodes per AOI event. The five most common AOIs during the study period were *P. jirovecii* pneumonia (43.4%), Cytomegalovirus disease (10.4%), *M. tuberculosis* (8.1%), wasting syndrome (8.1%), and Candidiasis (6.9%) (Additional file 1: Table S1). The prevalence of AOIs at presentation was 18.4%, and no decline was observed across groups (19.5% in period 1, 18.5% in period 2, and 17.3% in period 3; *P* = 0.74) (Table 1). Within the study period, the overall proportion of patients with an AOI was 21.0%, and no decline was observed across groups (22.5% in period 1, 21.0% in period 2, and 19.6% in period 3; *P* = 0.60). No significant difference was noted in the occurrence of the first AOI event among the three periods (log-rank test, *P* = 0.59; Fig. 1a).

By stratifying the AOI events (including repeated events) in each study period according to the interval since enrollment (≤90 days, 91–180 days, and ≥ 181 days; Fig. 1b), 91.7% of the events developed within 90 days, and among the three study periods, the proportion of events within each of the three intervals did not significantly differ (*P* = 0.11). Logistic regression results revealed that age [adjusted OR (AOR) per 10-year increase, 1.95; 95% CI, 1.58–2.41], heterosexual contact (vs. homosexual contact, AOR, 1.83; 95% CI, 1.05–3.19), and a CD4 cell count of < 200 cells/μL (vs. CD4 cell count ≥500 cells/μL at presentation, AOR, 40.84; 95% CI, 12.59–132.49) were associated with an AOI within 90 days of enrollment (Table 2).

**Table 2:** Univariable and multivariable analysis for factors predicting AOIs within 90 days of initial HIV care

	Univariable analysis		Multivariable analysis^a^	
	OR (95% CI)	*P*	AOR (95% CI)	*P*
Age, per 10-year increase	2.35 (2.02–2.74)	< 0.001	1.95 (1.58–2.41)	< 0.001
Male sex	0.66 (0.26–1.71)	0.39	2.38 (0.62–9.16)	0.21
HIV transmission route				
Homosexual	1.00 (Reference)		1.00 (Reference)	
Heterosexual	3.61 (2.48–5.26)	< 0.001	1.83 (1.05–3.19)	0.034
Bisexual	2.55 (1.51–4.31)	< 0.001	1.95 (0.96–3.94)	0.065
IDU	3.99 (1.37–11.66)	0.011	1.57 (0.35–6.96)	0.56
Unknown	7.10 (1.57–32.02)	0.011	1.39 (0.21–9.17)	0.73
Period				
Period 1 (2010–2011)	1.00 (Reference)		1.00 (Reference)	
Period 2 (2012–2013)	0.87 (0.63–1.21)	0.41	0.87 (0.56–1.34)	0.52
Period 3 (2014–2015)	0.80 (0.56–1.13)	0.20	0.92 (0.58–1.46)	0.72
Chronic kidney disease	4.03 (0.81–20.1)	0.089	0.84 (0.77–9.17)	0.89
Diabetes mellitus	3.78 (1.65–8.68)	0.002	1.02 (0.29–3.57)	0.97
Subgroup of CD4 cell count at presentation				
CD4 count ≥500 cells/μL	1.00 (Reference)		1.00 (Reference)	
CD4 count 200–499 cells/μL	1.05 (0.29–3.78)	0.94	0.91 (0.25–3.33)	0.88
CD4 count < 200 cells/μL	49.54 (15.60–157.40)	< 0.001	40.84 (12.59–132.49)	< 0.001
HBsAg seropositivity	2.09 (1.38–3.16)	0.001	1.32 (0.76–2.29)	0.33
HCV seropositivity	2.30 (1.20–4.39)	0.012	2.07 (0.81–5.34)	0.13

*Abbreviations*: *AIDS* acquired immune deficiency syndrome, *AOI* AIDS-defining opportunistic illness, *AOR* adjusted odd ratio, *CI* confidence interval, *HBsAg* hepatitis B surface antigen, *HCV* hepatitis C virus, *OR* odd ratio, *IDU* intravenous drug users

^a^All variables in the univariate analysis were selected for subsequent multivariate analysis

Overall, the results indicated that the development of AOIs did not significantly differ among the study periods. Most AOIs developed shortly after enrollment, and age, heterosexual contact, and CD4 cell count < 200 cells/μL at presentation increased the risk of an AOI within 90 days.

### Trends in AOI-related mortality and all-cause mortality

Of the 1264 enrolled patients, 1132 (89.6%) had survived, 56 (4.4%) had died, and 76 (6.0%) were lost to follow-up at the end of the study. The all-cause mortality within the study period was 4.4% [Total numbers: 56; AOI-related etiologies: 47 (83.9%), non-AOI-related infection: 4 (7.1%), liver disease: 3 (5.4%), cardiovascular disease: 2 (1.8%), and trauma: 1(1.8%)], and no decline was observed across groups (4.3% in period 1, 5.7% in period 2, and 3% in period 3, *P* = 0.15; Table 1). Within the entire study period, the overall proportion of AOI-related mortality was 3.7%; it did not significantly decreased from 3.3% in period 1 to 4.7% in period 2, and to 3.0% in period 3 (*P* = 0.38; Table 1). The overall mortality of each AOI event was 16.9%; it did not significantly improve across the three study periods (*P* = 0.82; Fig. 1b).

**Fig. 2:**
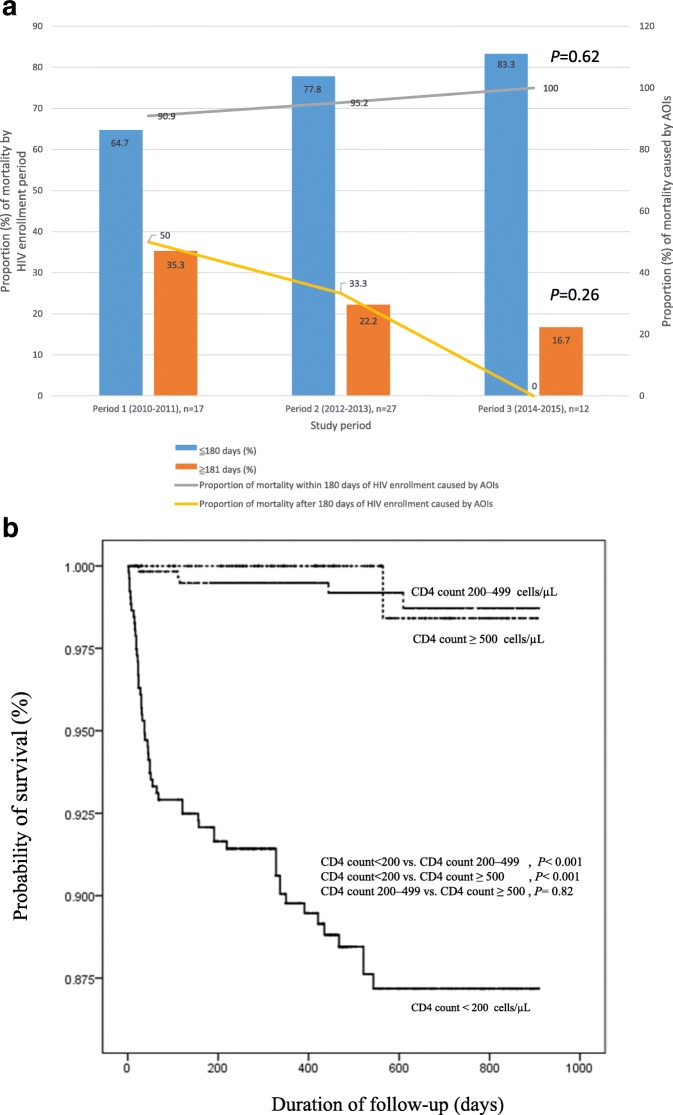
Analysis of AOI-related mortality. **a** Proportions of all-cause mortalities by interval since HIV enrollment and the proportion of AOIs as the cause of mortality by interval since HIV enrollment in the three study periods. Overall, 42 of the 56 deaths (75%) occurred within 180 days of enrollment for HIV care, and the proportion of mortalities within each of the two intervals did not significantly differ among the three study periods (*P* = 0.469). AOIs were the cause of 83.9% (47/56) of mortalities. The proportion of mortalities caused by AOIs declined significantly [from 95.2% (40/42) for deaths ≤180 days after HIV enrollment to 50.0% (7/14) for deaths ≥181 days after HIV enrollment (*P* < 0.001)]. As the cause of death stratified by interval since HIV enrollment, the proportion of mortalities caused by AOIs did not significantly differ among the three study periods (*P* = 0.620 and 0.264 for deaths ≤180 days and ≥ 181 days after enrollment, respectively). **b** Kaplan–Meier curves of all-cause mortality by CD4 cell count at presentation. The curves illustrated poorer survival among patients with a CD4 cell count of < 200 cells/μL at presentation compared with those with a CD4 cell count of 200–499 cells/μL at presentation (log-rank test, *P* < 0.001) and ≥ 500 cells/μL at presentation (log-rank test, *P* < 0.001)

When all 56 all-cause deaths in each study period were stratified by interval since HIV enrollment (≤180 days and ≥ 181 days; Fig. 2a), it was revealed that 75% of the deaths occurred within 180 days of enrollment, and the proportions of deaths within each of the two intervals did not differ significantly among the three study periods (*P* = 0.47, Fig. 2a). AOIs accounted for 95.2 and 50.0% of overall all-cause deaths ≤180 days and ≥ 181 days after enrollment for HIV care, respectively. The proportion of AOI as the cause of death stratified by interval since enrollment for HIV care did not significantly differ among the three study periods (*P* = 0.62 and 0.26 for AOI(s) as the cause of mortality within 180 days and after 180 days of enrollment for HIV treatment, respectively; Fig. 2a).

Univariable analysis results revealed that the HR for all-cause mortality in patients with a CD4 count of < 200 cells/μL at presentation was 16.86 (95% CI, 2.33–122.04) compared with those with a CD4 count of ≥500 cells/μL at presentation (Fig. 2b, Table 3). In the multivariable analysis, a CD4 count of < 200 cells/μL at presentation (vs. a CD4 cell count of ≥500 cells/μL at presentation) remained significantly associated with an elevated risk of all-cause mortality [adjusted HR (AHR), 11.03; 95% CI, 1.51–80.64; Table 3]. Other factors associated with an elevated risk of all-cause mortality were age (AHR per 10-year increase, 1.47; 95% CI, 1.21–1.77), heterosexual contact (vs. homosexual contact, AHR, 2.61; 95% CI, 1.32–5.16), unknown contact (vs. homosexual contact, AHR, 5.87; 95% CI, 1.27–27.08), and HBsAg seropositivity (AHR, 2.65; 95% CI, 1.42–4.94).

**Table 3:** Cox regression hazards model for factors predicting all-cause mortality in 1264 newly diagnosed HIV infection

	Univariable analysis		Multivariable analysis^a^	
	HR (95% CI)	*P*	AHR (95% CI)	*P*
Age, per 10-year increase	2.00 (1.71–2.32)	< 0.001	1.47 (1.21–1.77)	< 0.001
Male sex	1.09 (0.15–7.86)	0.93	3.67 (0.48–28.34)	0.21
HIV transmission route				
Homosexual	1.00 (Reference)		1.00 (Reference)	
Heterosexual	5.38 (3.03–9.55)	< 0.001	2.61 (1.32–5.16)	0.006
Bisexual	2.67 (1.03–6.90)	0.043	1.86 (0.71–4.88)	0.21
IDU	2.64 (0.36–19.38)	0.34	1.98 (0.26–14.98)	0.51
Unknown	11.14 (2.65–46.78)	0.001	5.87 (1.27–27.08)	0.011
Period				
Period 1	1.00 (Reference)			
Period 2	1.32 (0.72–2.43)	0.37	1.48 (0.80–2.76)	0.21
Period 3	0.71 (0.34–1.48)	0.35	0.92 (0.43–1.95)	0.82
Subgroup of CD4 cell count at presentation	17.59 (7.02–44.07)	< 0.001		
CD4 count ≥500 cells/μL	1.00 (Reference)		1.00 (Reference)	
CD4 count 200–499 cells/μL	1.29 (0.15–11.03)	0.82	1.21 (0.14–10.42)	0.86
CD4 count < 200 cells/μL	16.86 (2.33–122.04)	0.005	11.03 (1.51–80.64)	0.018
HBsAg seropositivity	3.30 (1.80–6.05)	< 0.001	2.65 (1.42–4.94)	0.002

*Abbreviations*: *AHR* adjusted hazard ratio, *AOIs* AIDS-defining opportunistic illnesses, *CI* confidence interval, *HBsAg* hepatitis B surface antigen, *HCV* hepatitis C virus, *HR* hazard ratio, *IDU* intravenous drug users

^a^All variables in the univariate analysis were selected for subsequent multivariate analysis

No significant differences in all-cause mortality and AOI-related mortality were observed among the study periods. Throughout all study periods and especially within 180 days of enrollment, AOIs remained the main cause of death. Age, a CD4 count of < 200 cells/μL at presentation, heterosexual and unknown contact, and HBsAg seropositivity were independent risk factors for all-cause mortality.

## Discussion

Throughout the three study periods, no significant differences were observed in the prevalence of AOI-related morbidity and mortality in patients with newly diagnosed HIV. AOI events were generally diagnosed at or shortly after presentation and enrollment (Figs. 1a, b). Throughout the study periods, particularly within 180 days of enrollment (95.2%; Fig. 2a), AOIs remained the main cause of death. The findings reveal that in Taiwan, in this era of contemporary HAART, AOI-related morbidity and mortality continue to pose a major challenge to patients with HIV at presentation or shortly after receiving a diagnosis of HIV.

Consistent with the Collaboration of Observational HIV Epidemiological Research (COHERE) study [14], the present study revealed that late presentation of HIV (CD4 count < 200 cells/μL at presentation) was associated with increased AOI-related morbidity and mortality (Tables 2 and 3). Late presentation has negative consequences not only for the patient but also for the general population. These include increased transmission of HIV to sexual partners [33, 34] and an increased demand for medical resources [15, 35]. Although several strategies for improving HIV care, including antimicrobial prophylaxis against *Pneumocystis jiroveci* pneumonia and *Mycobacterium avium* complex, have been adopted in Taiwan [23], further interventions to identify patients with HIV and initiate care earlier is urgently required to reduce AOI-related morbidity and mortality.

The substantial proportion of patients with late presentation HIV infection (CD4 count < 200 cells/μL at presentation) in this study (Table 1) is consistent with findings in studies conducted in both developing [12, 13] and developed [14–18] countries. In Taiwan, several active HIV surveillance programs have been adopted among specific populations, including blood and organ donors in 1988, military draftees in 1989, prison inmates in 1990, pregnant women in 2005, and people with sexually transmitted diseases and intravenous drug users (IDUs) in 2008. In 1997, a nationwide program of free anonymous VCT for HIV among high-risk populations was initiated at several hospitals, clinics, and nongovernmental organizations. It is unclear why the proportion of late-presenting patients did not change over the three study periods despite the implementation of active surveillance. Moreover, the proportion of patients with AIDS at presentation (37.7%) in this study was even higher than that reported (15%) during 1984–2005 in a previous nationwide cohort study [26]. In that study, the proportion of patients that received an AIDS diagnosis within 1 month of an HIV diagnosis was 21.1% in the pre-HAART (January 1, 1984 to March 31, 1997), 26.% in the early HAART (April 1, 1997 to December 31, 2001), and 10.9% in the late HAART eras (January 1, 2002 to December 31, 2005) [26]. The low proportion of such diagnoses during the late HAART era may have resulted from a rapid increase in IDUs with HIV infections (from 1.7% in 2002 to 68.6% in 2006) [36, 37], most of whom were diagnosed through active surveillance in the penal system [26, 38]. However, HIV infection in IDUs has consistently decreased since the Taiwan CDC launched harm reduction programs in August 2005 [39]. Starting in 2008, the epidemic changed, with infections mainly occurring through sexual transmission, especially among men who have sex with men (MSM), as reported in other Asian countries [40, 41]. The higher rate of AIDS at presentation observed in the present study compared with that in previous studies [26] may have resulted from a growing population of MSM who are unable to access HIV testing and subsequent care due to the dual stigma and discrimination of both homosexuality and HIV [42]. Therefore, to alleviate the recent surge of late HIV presentation among MSM, investigating the risks associated with late presentation and adopting consequent interventions in this high-risk population should be a priority.

The early diagnosis of patients with HIV infection is challenging; however, Taiwan presents various opportunities for the implementation of interventions. The first opportunity is provided by surveys of trends in late presentation in known risk groups to identify those at increased risk of late presentation [12, 13, 18, 43, 44]. The second opportunity involves identifying barriers to HIV testing and subsequent care in groups at increased risk of late presentation. The present guidelines recommend that sexually active homosexual and bisexual men get tested for HIV at least annually [45]. In many Asian countries, the estimated frequency of testing in those high-risk populations is below the recommended level of 60–80%, which is crucial for reducing the incidence of HIV in a population [41, 46]. Although the proportion of HIV infections diagnosed through VCT programs increased from 33.4% in period 1 to 43.0% in period 3 (Table 1), almost half of the new diagnoses were not made through active surveillance for HIV infection. Efforts to identify social [47–49], psychological [13], geographical [13], and economic barriers [13] that prevent high-risk groups from receiving regular HIV testing or timely medical care [17] should be increased. The third opportunity involves adopting novel approaches to HIV testing. In addition to facility-based testing, which is widely used in Taiwan, flexible approaches such as peer-led [50], indicator condition-guided [51], and home-based [52] testing should be targeted by incorporating the current knowledge of HIV prevalence and the causes of late presentation. For example, in Taiwan, rapid oral HIV testing by IDUs after release from the criminal justice system has been accepted [53]. The fourth opportunity for intervention is educating primary physicians on the early presentation of HIV infection and acute retroviral syndrome. Primary physicians may not be sufficiently sensitive to the indicators of such HIV-associated conditions or the symptoms of acute retroviral syndrome that cause patients with HIV to seek medical care [54]. In this study, no significant decline in the proportion of HIV diagnoses at an acute HIV status was observed over the three study periods (Table 1). This might be reversed by increasing the awareness of primary-care physicians.

The key strength of this study is that it is the first in this era of contemporary HAART to analyze the current trends in AOI-related morbidity and mortality in newly diagnosed patients with HIV infection in Taiwan after the implementation of harm reduction programs in August 2005. The findings are critical for public health policies in terms of regulating HIV control and may be generalizable to other Asia-Pacific [40, 41] and North American countries [55] with similar HIV epidemics among the MSM population who may be unable to access HIV testing due to stigma and discrimination of both homosexuality and HIV [42]. However, our research had several limitations. First, the results may not be generalizable to the entire population of Taiwan. During the study period, 12,786 patients with newly diagnosed HIV were registered with the Taiwan CDC. Of these, 9.9% (1264/12,786) were enrolled in this study. Most of the participants (97%) were men, and the three main transmission routes were MSM (78.5%), heterosexual contact (13.4%), and intravenous drug use (3.7%) [56–61]. Although the sex distribution and HIV transmission routes are similar to those in the present study, our findings must be confirmed through nationwide surveillance. Second, there is no single definition of late presentation with HIV infection [62]; previously used definitions considered different CD4 cell counts (in the range of < 50 to < 350 cells/μL) [14, 15, 63]. In this study, late presentation was defined as a CD4 cell count of < 200 cells/μL at presentation. Therefore, the prevalence of late presentation in this study may not be comparable to that in other studies. Finally, 94 patients were excluded from the present study because they visited the outpatient clinic only once and were lost to follow-up during the observation period. Because the etiologies of patients lost to follow-up could not be determined, the effect on the prevalence of late presentation and AOI-related morbidity and mortality remains unknown. However, a comparison of the 1264 enrolled patients and 94 patients excluded due to being lost to follow-up revealed no significant differences between these two groups with respect to sex, age, and risk of HIV transmission.

## Conclusion

Although considerable progress in reducing the burden of HIV and AOI-related morbidity and mortality has been made in Taiwan since the early 1980s, further improvements are warranted. The prevalence and timing of AOIs remain a critical challenge in delivering early HIV care. Nationwide surveillance to investigate the trends in late presentation among different risk groups and analyze the associated risks is urgently required. Improved surveillance is warranted for implementing effective, targeted interventions to improve early diagnosis of HIV infection and further reduce AOI-related morbidity and mortality.

## Additional file

Additional file 1: **Table S1.** The spectrum of 394 AIDS-related opportunistic illnesses and the distribution of the median CD4+ lymphocyte. (DOCX 20 kb)

## Abbreviations

AIDS: Acquired immune deficiency syndrome
AOI: AIDS-related opportunistic illnesses
CI: Confidence interval
DM: Diabetes mellitus
HAART: Highly active antiretroviral therapy
HBsAg: Hepatitis B surface antigen
HCV: Hepatitis C virus
HIV: Human immunodeficiency virus
HR: Hazard ratio
IDU: Intravenous drug users drug users
OR: Odds ratio
PY: Person-year
SD: Standard deviation
VCT: Voluntary counseling and testing
VL: Viral load
