# Supplementary material for: AIDS-related opportunistic illnesses and early initiation of HIV care remain critical in the contemporary HAART era: a retrospective cohort study in Taiwan
Source: BMC Infect Dis. 2018 Jul 28;18:352. doi: 10.1186/s12879-018-3251-1 (PMC6064097; doi:10.1186/s12879-018-3251-1)
Supplement: Supplementary file 1 — Table S1. The spectrum of 394 AIDS-related opportunistic illnesses and the distribution of the median CD4+ lymphocyte. (DOCX 20 kb) [file 12879_2018_3251_MOESM1_ESM.docx]

Table S1 The spectrum of 394 AIDS-related opportunistic illnesses and the distribution of the median CD4+ lymphocyte

|  | | No. of case (% of total cases), n=394 | Median CD4^+^ T cell, cell/µL (IQR) |
| --- | --- | --- | --- |
| Opportunistic infection | | 372 (94.5) |  |
|  | *Pneumocystis jirovecii* pneumonia | 171 (43.4) | 25 (44) |
|  | Cytomegalovirus disease (other than liver, spleen, or nodes) | 41 (10.4) | 20 (49) |
|  | *Mycobacterium tuberculosis* | 32 (8.1) | 55 (171) |
|  | Wasting syndrome | 32 (8.1) | 50 (95) |
|  | Candidiasis (esophagus, bronchi, trachea, lung) | 27 (6.9) | 33 (83) |
|  | Cryptococcosis, extrapulmonary | 24 (6.1) | 33 (41) |
|  | Disseminated *Mycobacterium avium* complex infection or *M. kansasii* | 20 (5.1) | 39 (43) |
|  | Cryptosporidiosis, chronic intestine | 4 (1.0) | 28 (34) |
|  | HIV encephalopathy | 6 (1.5) | 162 (447) |
|  | HSV, chronic ulcer greater than 1 month; or bronchitis, pneumonitis, or esophagitis | 5 (1.3) | 67 (137) |
|  | Salmonellosis septicemia, recurrent | 4 (1.0) | 25 (33) |
|  | Recurrent pneumonia | 2 (0.5) | 78 (N/A) |
|  | Progressive multifocal leukoencephalopathy | 2 (0.5) | 134 (N/A) |
|  | *Toxoplasma* encephalitis | 1 (0.3) | 53 (0) |
|  | *Penicillium marneffei*, disseminated or extrapulmonary | 1 (0.3) | 20 (0) |
|  | Histoplasmosis, disseminated or extrapulmonary | 0 (0) | N/A |
|  | Coccidioidomycosis, disseminated or extrapulmonary | 0 (0) | N/A |
|  | Isosporiasis, chronic intestine (greater than one month) | 0 (0) | N/A |
| Opportunistic malignancy | | 22 (5.5) |  |
|  | Kaposi’s sarcoma | 10 (2.5) | 138 (242) |
|  | Lymphoma | 12 (3.0) | 96 (124) |
|  | Invasive cervical cancer | 0 (0) | N/A |

Abbreviations: HIV, human immunodeficiency virus; HSV, herpes simplex virus; IQR, interquartile range; N/A, not applicable.
